# Investigation of bacterial diversity in *Cajanus cajan*-planted gangue soil via high-throughput sequencing

**DOI:** 10.1080/21655979.2021.1976043

**Published:** 2021-09-21

**Authors:** Shiming Han, Yuexia Wang, Yuan Li, Kaiyi Shi

**Affiliations:** a School of Biological Science and Technology, Liupanshui Normal University, Liupanshui Guizhou, China; b Human Resources Office, Liupanshui Normal University, Liupanshui Guizhou, China; c Guizhou Coal Product Quality Supervision & Inspection Institute, Liupanshui Guizhou, China; d School of Chemistry and Chemical Engineering, Qiannan Normal University for Nationalities, Duyun, Guizhou, China; e School of Chemistry and Materials Engineering, Liupanshui Normal University, Liupanshui, Guizhou, China

**Keywords:** *Cajanus cajan*, gangue, principal component analysis, high-throughput sequencing, restoration

## Abstract

The ecological restoration of coal gangue can be achieved by planting *Cajanus cajan* (pigeon pea) because of its developed root system. The close relationships soil microorganisms have with plants are crucial for improving soil composition; the soil composition affects nutrient absorption. The microbial composition and function of soil planted with *C. cajan* in reclaimed land were compared with soil that was not planted with *C. cajan* (the control). Results showed that the dominant microflora in the soil significantly changed after planting *C. cajan*. Before planting, the dominant microflora included members of the phyla Sulfobacteria and Acidobacteria. After planting, the dominant microflora contained bacteria from phyla and classes that included *Actinobacteria, Acidimicubia, Thermoleophilia*, and *Anaerolineae*. Additionally, there were significant differences in the bacterial composition of each layer in soils planted with *C. cajan*. Principal component analysis revealed that the interpretation degrees of the results for PC2 and PC3 axes were 10.46% and 3.87%, respectively. The dominant microflora were Vicinamibacterales, *Nocardioides*, and *Arthrobacter* in the surface soil; *Actinophytocola* and *Sphingomonas* in the deep soil; and *Sulfobacillus* and *Acidimicrobium* in the mixed-layer soil. Function prediction analysis using the bioinformatics software package PICRUSt revealed that the abundance of operational taxonomic units corresponding to sigma 54-specific transcriptional regulators, serine threonine protein kinase, and histidine kinase increased by 111.2%, 56.8%, and 47.4%, respectively, after planting *C. cajan*. This study provides a reference for interactions among microorganisms in reclaimed soils for guiding the development and restoration of waste coal gangue hills.

## 1. Introduction

Gangue, which is a waste product of global coal and carbon energy industry, has the properties of easy acidification with high sulfur content [1]. The stock of coal gangue in China has reached the level of 1 billion and is increasing at a rate of 200 million per year [2]. Thus, the restoration of coal gangue is a concern of the government and scholars. The contents of organic matter, nitrogen, phosphorus, and potassium in coal gangue are extremely low, which makes it difficult for plants to use it for growth and ecological recovery [3]. Microorganisms play an essential role in plant growth and environmental pollutant degradation [4]. Studies have demonstrated that under adverse conditions, bacteria, fungi, and other microbes with mycorrhiza can expand the scope of a plant’s root system, thereby helping plants absorb and activate soil nutrients [5]. In this manner, plant phosphorus deficiency, nutritional status, cold resistance, drought resistance, salt tolerance, and resistance to heavy metal pollution can be improved [6]. Meanwhile, the growth and activity of rhizobium is promoted in addition to the plants’ immunity to disease [7]. Therefore, planting specific plants on gangue hills is a key procedure in the ecological reconstruction of gangue hills to utilize the life activities and technologies of microorganisms and plant rhizosphere microorganisms. This would improve soil substrates [8] and help excavate the potential fertility of gangue hills to accelerate vegetation restoration and promote sustainable development of the ecosystem [9].

Soil is the habitat of many microorganisms, and its physical/chemical properties as well as nutrient composition determine the species, both in quantity and diversity. The succession of the soil microbial community reflects trends in the soil texture and quality, which have become an important indicator of soil restoration performance evaluation. Soil microbial diversity refers to changes in soil microorganisms at the level of heredity, species, and ecology. The most basic and essential reflection of microbial diversity is the genetic diversity. The genetic material and gene expression of microorganisms among different populations show obvious differences, including the composition of amino acids and enzymes. Species diversity is the most direct expression of soil microbial diversity, referring to the richness and evenness of microorganisms in a soil ecosystem.

In recent years, research into microbial remediation in gangue hills has primarily focused on the soil fixation of reclaimed plant roots, physical and chemical properties of reclaimed soil, and enzyme activities of plant soil. Dangi et al. [10] studied changes in soil microbial concentration and community structure in the mining areas of northeastern Wyoming, USA, and revealed a significant relationship between vegetation and microbial community between different vegetation types and reclamation periods [10]. In addition, 11,analyzed the effect of root spatial distribution and properties on the behavior of soil resistance around tree stems and found that roots were evenly distributed in a 0.7 m radius around the main stem. 12,studied the planting management modes in a forest [Shaanxi Province, China), including the physical and chemical properties of soil organic matter, total nitrogen, and rapidly available potassium, and found linear correlations among soil organic matter, total nitrogen, available phosphorus, and rapidly available potassium with different management modes.

Microorganisms play an important role in plant growth. 13,separated and purified dominant bacterial strains from wheat *rhizosphere* soil and found that the superior bacterial strain X20 was *Bacillus*, which is a potential inhibitor for developing bacterial-based bioherbicides for wild oats weed control management in wheat fields. Although a close relationship exists between soil microorganisms and plants, few reports exist regarding the microbial diversity of plant soil. It is, therefore, necessary to study the composition, function, and evolution of soil microorganisms in reclaimed mining areas.

The composition of microorganisms in soil can be obtained using the gene sequencing technology. The Sanger method of the first-generation sequencing technology is based on DNA synthesis reaction. This technology has a long reading length and high accuracy, yet the flux is not high. The second-generation sequencing technology represented by the Illumina platform realizes high-throughput sequencing, achieves large-scale parallel sequencing, and promotes the development of genomics in life science fields. This technology has the advantages of a short reading length, high flux, and high accuracy.

In this study, the reclamation demonstration base of a gangue hill in Liupanshui was evaluated and the microbial diversity of soil at the different depths of reclaimed plants was systematically analyzed using high-throughput sequencing. The functions of related microorganisms were predicted to provide a theoretical basis for the interaction mechanism between the reclaimed plants and gangue. The research results provide a reference for the development of waste coal gangue hills and restoration of polluted mining areas.

## 2. Materials and methods

### 2.1 Overview of gangue hill reclaimed demonstration site

Panzhou Juneng Coal Preparation Co., Ltd. (Liupanshui Pan City, Xiangshui Town, Che Tian Village, Lijiagou] has a demonstration site of coal gangue planted with *C. cajan*. The geo coordinates of reclamation is 25°25ʹ54 ‘N, 104°36ʹ7’ E, 1,390 m above sea level. In 2018, the Chinese government performed the reclamation tests of coal gangue dumps by planting *C. cajan*. The different sources of *C. cajan* from Yunnan, Guizhou, Sichuan, Guangxi, Hunan, and Jiangxi were planted, and the demonstration areas were approximately 13,000 m^2^ in total. When the *C. cajan* plants grow to a height of 2.5–3 m, the root system develops and grows up to 0.70 m into the soil layer below. The root system is rich in nodules and resistant to infertile soil.

### 2.2 Collection and treatment of soil samples

On 25 July 2020, soil samples were collected from Panzhou Juneng Coal Preparation Co., Ltd. A five-point sampling method was adopted, and 10 g of top soil (labeled as mt001, mt002, and mt003), mixed-layer soil (from 0.20–0.30 m depth, labeled as mgm1, mgm2, and mgm3), and deep soil (0.50–0.70 m depth, labeled as mt201, mt2002, and mt203) were collected in triplicate. As controls, soils without *C. cajan* were also collected, and the soil layer samples were labeled as nt1, nt2, and nt3, respectively, whereas the samples of mixed-layer soil and gangue were labeled as ngt1, ngt2 and ngt3. Large soil pieces were removed, and samples were placed in bags for transportation to the laboratory at −80°C in a refrigerator. Two gram of each sample was collected to remove fine plant roots and mycelium impurities, and the remaining samples were stored at −80°C in a refrigerator. The soil bacterial community was determined using high-throughput sequencing, which was performed at the Majorbio Bio-Pharm Technology Co. Ltd. (Shanghai, China).

### 2.3 DNA extraction and polymerase chain reaction (PCR) amplification

Microbial community genomic DNA was extracted from *C. cajan* root samples using the E.Z.N.A.® soil DNA Kit (Omega Bio-tek, Norcross, GA, U.S.) according to the manufacturer’s instructions. DNA integrity was checked on 1% agarose gel, whereas the DNA concentration and purity were determined using NanoDrop 2000 UV–Vis spectrophotometer (Thermo Scientific, Wilmington, USA). The hypervariable region V3–V4 of the bacterial 16S rRNA gene was amplified with the primer pairs 338 F (5′-ACTCCTACGGGAGGCAGCAG-3′) and 806 R (5′-GGACTACHVGGGTWTCTAAT-3′) using the ABI GeneAmp® 9700 PCR thermocycler (ABI, CA, USA). The PCR amplification of the 16S rRNA gene was performed as follows: initial denaturation at 95°C for 3 min, followed by 27 cycles of denaturation at 95°C for 30 s, annealing at 55°C for 30 s, extension at 72°C for 45 s, single extension at 72°C for 10 min, and end at 4°C. The PCR mixtures contained 4 μL 5× *TransStart* FastPfu buffer, 2 μL 2.5 mM dNTPs, 0.8 μL forward primer (5 μM), 0.8 μL reverse primer (5 μM), 0.4 μL *TransStart* FastPfu DNA Polymerase, 10 ng template DNA, and up to 20 μL H_2_O. PCR was performed in triplicate. The PCR product was extracted from 2% agarose gel and purified using the AxyPrep DNA Gel Extraction Kit (Axygen Biosciences, Union City, CA, USA) according to the manufacturer’s instructions and quantified using Quantus™ Fluorometer (Promega, USA) [14].

### 2.4 Illumina MiSeq sequencing

Purified amplicons were pooled in equimolar and paired-end sequenced on the Illumina MiSeq PE300 platform/NovaSeq PE250 platform (Illumina, San Diego, USA) according to the standard protocols established by Majorbio Bio-Pharm Technology Co. Ltd.

### 2.5 Sequencing data processing

Raw 16S rRNA gene sequencing reads were demultiplexed [15], quality-filtered by fastp version 0.20.0, and merged by FLASH version 1.2.7 using the following criteria [16]: (i) the 300 bp reads were truncated at any site receiving an average quality score of <20 over a 50 bp sliding window, whereas truncated reads <50 bp and reads containing ambiguous characters were discarded; (ii) only overlapping sequences >10 bp were assembled according to their overlapped sequence. The maximum mismatch ratio of overlap region was 0.2. Reads that could not be assembled were discarded; and (iii) samples were distinguished according to the barcode and primers, the sequence direction was adjusted, exact barcodes were matched, and two nucleotide mismatches were detected during primer matching.

Operational taxonomic units (OTUs) with 97% similarity cutoff were clustered using UPARSE version 7.1 [17], and chimeric sequences were identified and removed. The taxonomy of each OTU representative sequence was analyzed using RDP Classifier version 2.2 against the 16S rRNA database (e.g., Silva v138) and ITS1 rRNA database (unite8.0/its_fungi) using a confidence threshold of 0.7 [18].

## 3. Results and discussion

Different samples (planted and unplanted *C. cajan* soils at different depths) were analyzed. Coverage, Shannoneven, Sobs index were used to reflect community evenness, diversity, and richness based on α-diversity analysis. Microbial community composition, dominant microflora, and interaction with environment factor were detected using composition analysis and principal component analysis (PCA). A Kruskal–Wallis *H* test was performed on species within microbial colonies in the different depths of soil planted with *C. cajan*. In the end, the annotation information of OTUs at the COG ((Clusters of Orthologous Groups)) function level (corresponding to enzymes) and abundance information of each function were explored.

### 3.1 Sequencing data statistics

Via the DNA sequencing of microorganisms from 15 samples, a total of 39,1959,579 bp were detected. The sequence lengths of the 15 samples and related information are shown in Table 1. The shortest sequence was 203 bp in length in the coal gangue sample nt1 (Table 1). The longest sequence was 531 bp, and the average sequence length in the mixed soil samples of the middle layer was 414.8 bp.

**Table 1.** Sample information statistics

Sample Info	Seq_num	Base_num	Mean_length	Min_length	Max_length
mgm1	51,890	21,506,887	414.4707	223	477
mgm2	52,988	22,026,210	415.683	385	503
mgm3	59,434	24,985,673	420.3936	355	506
mt001	69,946	29,181,912	417.2063	203	495
mt002	70,808	29,519,893	416.9005	233	509
mt003	72,666	30,117,555	414.4656	239	483
mt201	70,075	29,045,300	414.4888	223	509
mt202	67,361	27,956,258	415.0214	253	509
mt203	58,409	24,227,805	414.7958	240	531
ngt1	65,980	27,581,195	418.0236	317	526
ngt2	62,802	26,134,467	416.1407	253	464
ngt3	54,130	22,299,332	411.9588	250	431
nt1	73,377	30,245,912	412.1988	203	516
nt2	53,641	22,002,708	410.1845	277	462
nt3	61,416	25,128,472	409.1519	219	461
**Total**	**944,923**	**391,959,579**	**6221.084**	**3873**	**7382**
Note: The sample name in the first column and sample-related information in the second to sixth columns are sequence number, base number, average length, shortest sequence length, and longest sequence length, respectively.					

### 3.2 OTU species classification statistics

A total of 4,689 taxonomic clusters (OTUs) were identified from the 15 soil samples, including 1 domain, 1 kingdom, 42 phyla, 131 classes, 306 orders, 456 families, 871 genera, and 1,721 species. Among the six soil samples, mt001–mt203 had a relatively high diversity of microbial communities, the proportion of dominant bacteria was low, and the species distribution was relatively uniform. The species diversity of the microbial community in the soil samples mgm1–mgm3 and ngt1–ng3 was relatively low, and the proportion of dominant bacteria was relatively high.

### 3.3 Analysis of α-diversity of soil microbial community

**Figure 1.**
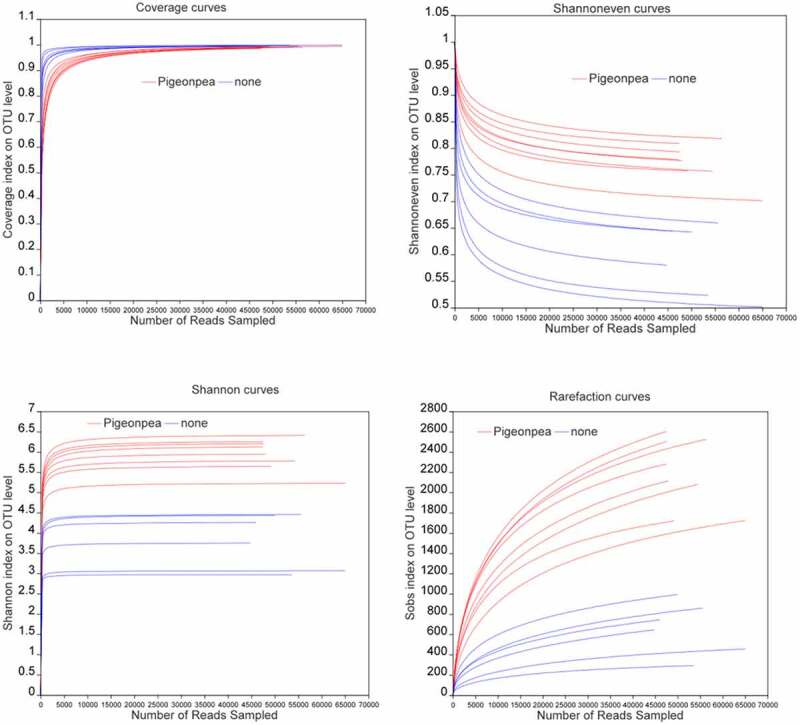
Alpha diversity index in planted and unplanted soils

The distribution of the *α*-diversity index of planted and unplanted soils is shown in Figure 1. The coverage index reflects community coverage, whereas the Shannoneven index reflects community evenness. The Shannon index reflects community diversity, whereas the Sobs index represents the richness of the microbial community in the environment compared with other organisms. As can be seen from the figure, when the sample reads reached 10,000, the coverage index of each sample was close to 1 and the curves tended to flatten, indicating that the sample sequencing volume was sufficient. With the increase in the number of reads, the Shannoneven index gradually decreased. When reads reached 15,000, the Shannoneven index of soil samples planted with *C. cajan* tended to be flat and the index values were all higher than those of unplanted soil samples, indicating that the community uniformity of the planted soil was superior. The Shannon and Sobs index charts showed that the community diversity and richness of soil planted with *C. cajan* was increased compared with unplanted soils.

### 3.4 Composition analysis of sample soil microbial community

**Figure 2.**
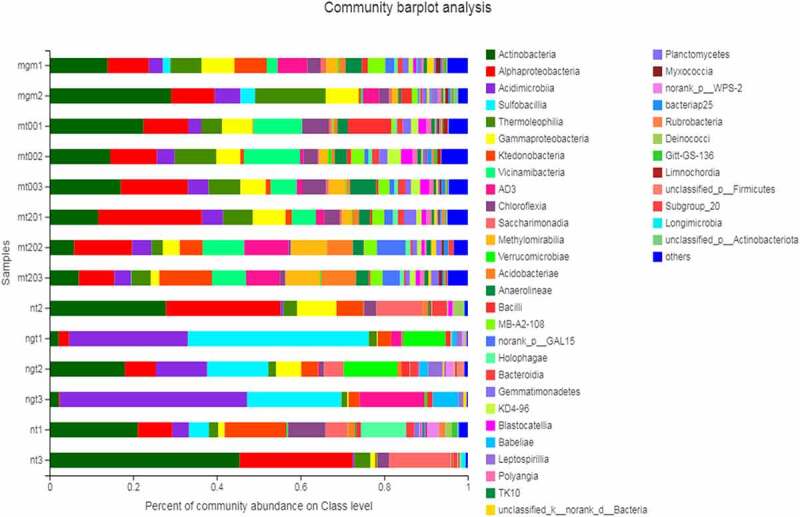
Horizontal community composition of microbiota

A total of 4,689 OTUs were obtained from 15 samples, which were divided into 42 phyla. Large differences were observed in the main community composition of the different soil samples and their relative abundance. Figure 2 shows the microbial community composition of each sample soil at the class level. The dominant microflora of the planted soil samples (mgm1–mt3) primarily included Actinobacteria, α-Proteobacteria, Acidimicrobiia, Thermoleophilia, Gammaproteobacteria, and Vicinamibacteria, accounting for 5.87%–29.5%, 8.46%–24.79%, 3.08%–6.19%, 2.71%–16.91% and 1.18% of the total sequence number, respectively. The dominant microflora in the unplanted reclaimed soil (ngt1–ngt3) consisted of *Acidimicrobiia* and sulfobacilli, accounting for 12.30%–44.73% and 14.61%–43.27% of the total microflora, respectively. However, *Actinobacteria* and α-*Proteobacteria* accounted for 21.09%–45.46% and 8.26%–27.41% of the total microflora in coal gangue samples (nt1–nt3). In the unplanted reclaimed soil samples, the number of OTUs of *Acidimicrobiia* was higher than that of the corresponding *C. cajan*-planted soil samples. *Sulfobacillus* is the dominant microflora in *C. cajan*-planted soil samples, but it accounted for only a small proportion in the unplanted reclaimed soil samples. In the reclaimed soil (mt001–mgm3), the proportion of actinomycetes, α-*Actinobacteria*, and *Acidimicrobiia* were smaller, but the community composition was more complex and the variety of classes was richer than the unplanted soil.

**Figure 3.**
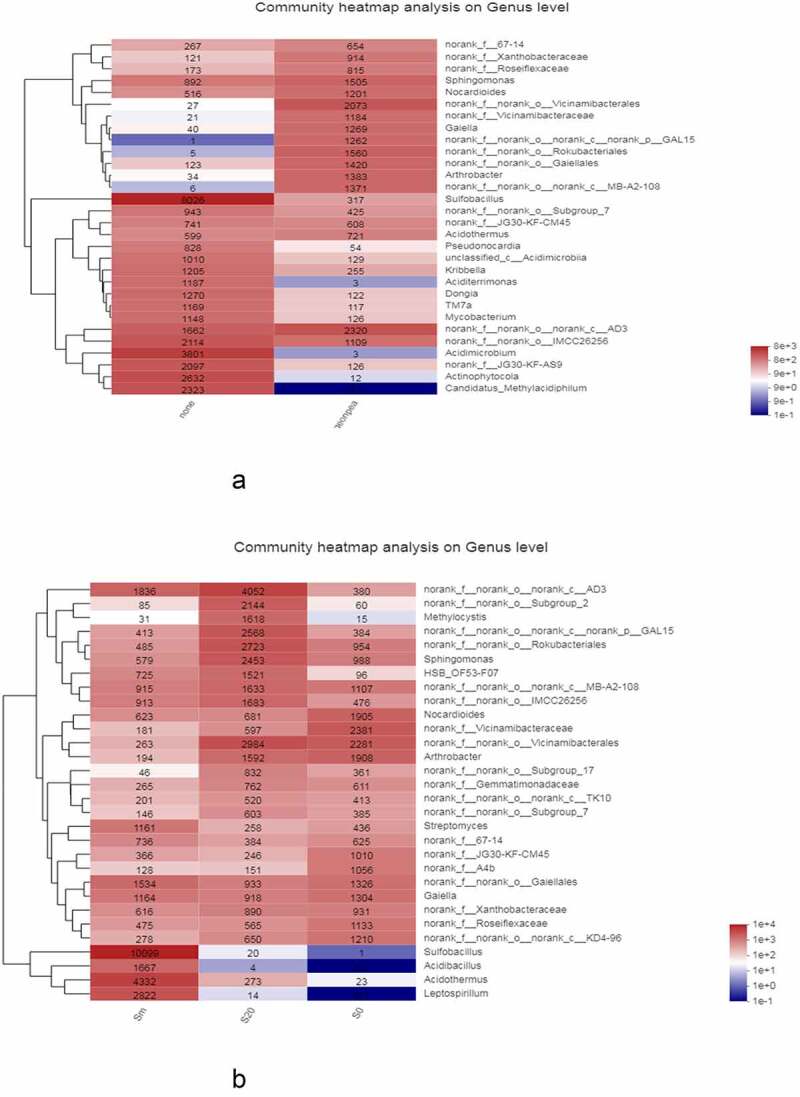
A) Heatmap of microorganisms at the genus level comparison between soils planted with and without *C. cajan*. B) Heatmap of microorganisms at the genus level of surface soil (S0), deep soil (S20), and gangue mixed layer (Sm) in soils planted with *C. cajan.*

To further understand the differences and similarities in the community composition of soil samples at the taxonomic level, the relative microbial abundance in each genus level of planted and unplanted soil was selected and a heatmap was constructed for comparative study (Figure 3a). The unplanted soil sample had a single microbial community composition and relatively few microbial species. With the exception of sulfobacilli, which accounted for the vast majority of microorganisms, other bacteria were much lower in content. The level of sulfobacilli in the soil samples planted with *C. cajan* was very low, whereas other bacteria were diverse and relatively uniform.

For planted soil, the composition of microorganisms at different soil depths was compared (Figure 3b). The soil microbial composition was varied at different depths. For example, the content of sulfobacilli was particularly high in the mixed layer of soil and gangue, lower at depths of ≤20 cm, and almost zero at the surface layer. *Acidibacillus, Acidothermus*, and *Leptospirillum* also showed a similar decreasing trend of spatial gradient. However, some other strains, including *Nocardioides, Norank_F_A4b*, and *Norank*_*F_Roseiflexaceae*, showed an opposite trend.

**Figure 4.**
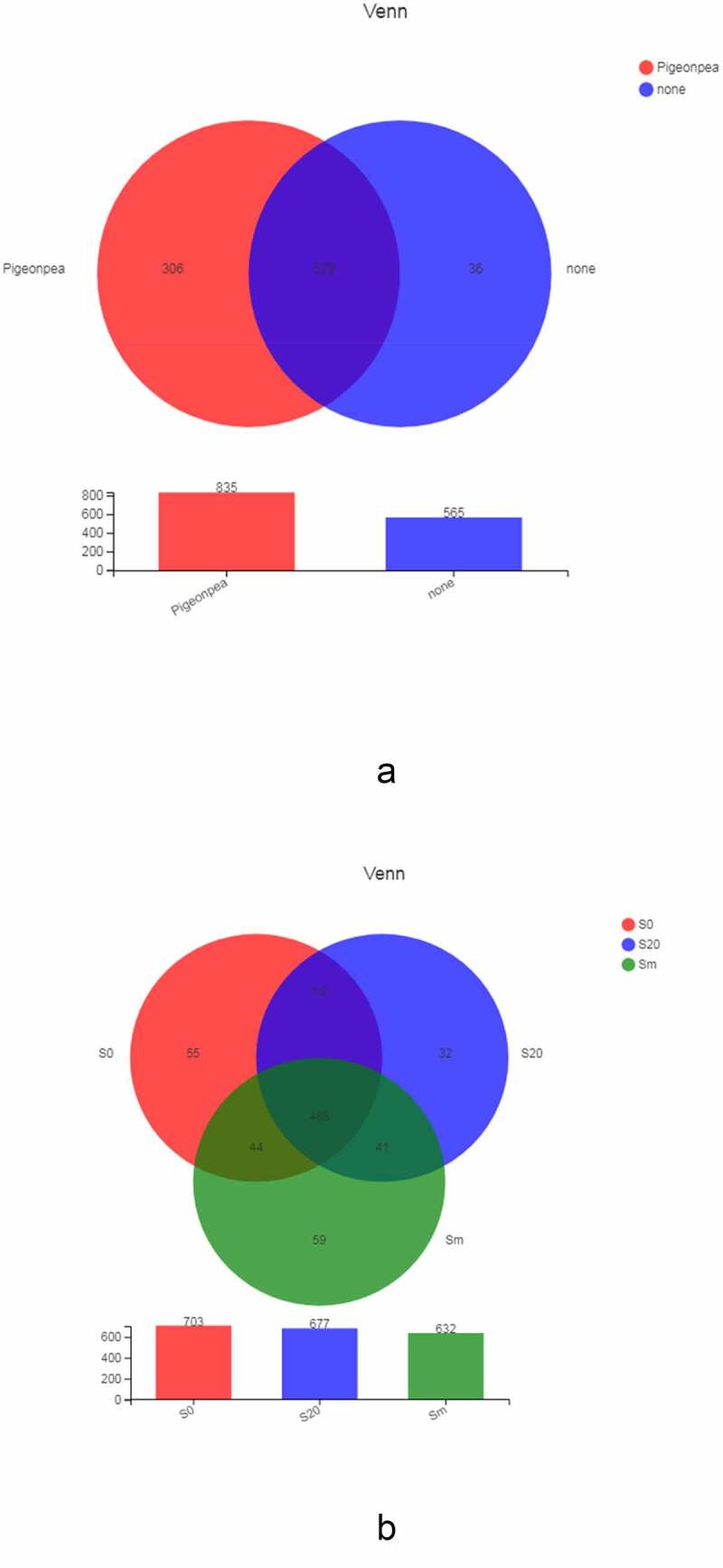
A) Venn diagram of the OTU distribution of bacteria in cultivated and unplanted soil. B) Venn diagram of the OTU distribution of soil bacteria at the different depths of soils planted with *C. cajan.*

OTUs with similar levels were selected for Venn chart analysis to reflect the common and unique OTU numbers of each treatment, and the commonalities and differences among samples were analyzed in detail. Figure 4a shows the bacterial composition between soil samples planted with *C. cajan* (mt001–mt003, mt201–mt203, and mgm1–mgm3) and unplanted soils (ngt1–ngt3 and nt1–nt3). The total number of OTUs in the planted and unplanted groups was 529, accounting for 63.35% and 93.63% of the total OTUs in their respective samples. The *C. cajan* group had 306 OTUs only, whereas the unplanted group had 36.

We compared the bacterial composition at different depths (surface layer, mixed layer, and deep layer) in the soils planted with *C. cajan* (Figure 4b). The total number of OTUs from the 3 groups was 488, which accounted for 69.42%, 72.08%, and 77.22% of the samples, respectively. These included 116 OTUs in the surface layer (S0) and deep layer (S20), 44 OTUs in the surface layer (S0) and the gangue mixed layer (Sm), and 41 OTUs in S20 and Sm.

**Figure 5.**
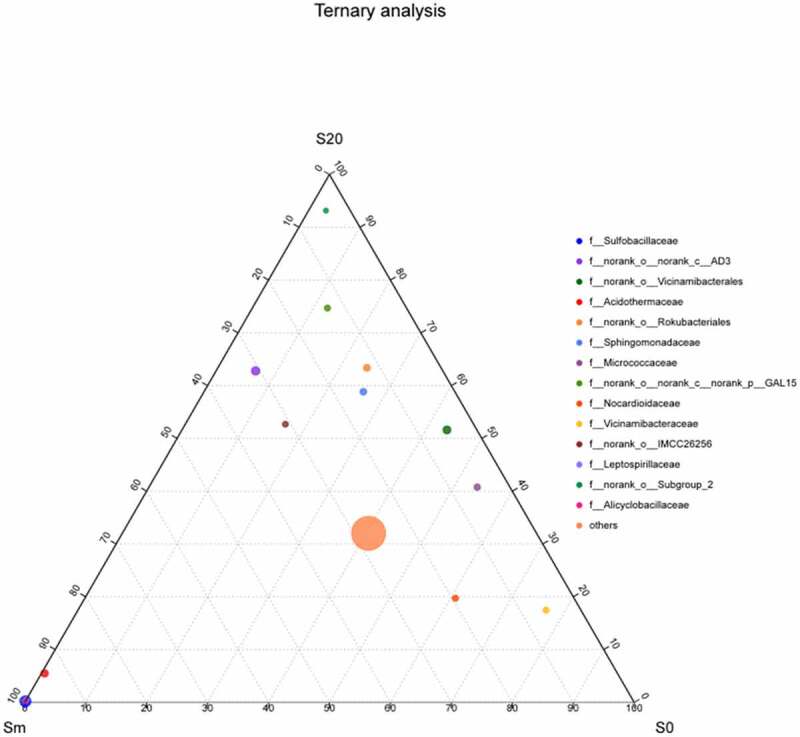
Ternary plot of microbial composition in soil at different depths

As per the taxonomic information of species, the species composition of microorganisms in the soil samples of the three groups planted at different depths at the family level was compared and analyzed and a ternary phase diagram was obtained (Figure 5). Sulfobacillaceae and Leptospirillaceae were detected on the top of the gangue mixed layer (Sm), indicating that these two bacteria exist in gangue and the overlying soil. Vicinamibacteria were primarily found in surface soil (S0) and deep soil (S20), accounting for 43.5% and 51.6% of bacteria, respectively. Acidothermaceae were located on the edge of S20 and Sm, near Sm, and had a high content in Sm, accounting for 94.0% and 5.5% in S20.

There were similarities and differences in the composition of bacterial communities in different soils at different classification levels. In all soil samples, *Actinobacteria* and α-*Proteobacteria* were relatively abundant, indicating that they exist widely in various soil environments and have the adaptive characteristics of growth and reproduction in the special environment of coal gangue. *Sulfobacillus* and *Acinetobacter* occupied a high proportion in reclaimed soil without *C. cajan*, yet the proportion in other samples was mostly small. This study found a significant difference between the microbial diversity of soils planted with *C. cajan* and unplanted soils, and the microbial diversity of planted soil was significantly higher than that of unplanted soil. In nine planted soil samples, Actinobacteria, α-Proteobacteria, Acidimicrobiia, Thermoleophilia, γ-Proteobacteria, and Anaerolineae were relatively abundant, whereas in the soil samples not planted with *C. cajan*, the abundant microflora consisted mostly of Actinobacteria, which can promote the decay of animal and plant remains in soil. **Abdoulaye *et al*. [38]** found that Actinobacteria can promote the growth and yield of corn.

Alphaproteobacteria perform the photosynthesis and metabolism of C1 compounds. Monteil et al. [19] reported that Alphaproteobacteria inhabiting the sediments and water column had the peculiarity of biomineralizing both intracellular magnetic particles and calcium carbonate granules. Tian et al. [20] reported that Alphaproteobacteria were the dominant class according to an analysis of microbial communities. Acidimicrobiia can perform iron oxidation and reduction reactions and have the ability to perform ore oxidation and synthesis of new active substances. They are abundant in diversified aquatic environments, including acidic mine water, wastewater sludge, fresh water, and marine habitats (21]. Thermophiles are characterized by high temperature resistance and are thus often abundant in hot springs and soil samples. Lin et al. [22] studied the microbial community that degrades the organic sulfur molecule dimethyl sulfonate and found that Gammaproteobacteria was the dominant strain. Anaerolineae are anaerobic bacteria that are often found in heavy metal-polluted environments [23]. Wu et al. [24] also detected Anaerolineae in polluted estuaries and pelagic sediments.

### 3.5 Beta-diversity analysis of bacteria

**Figure 6.**
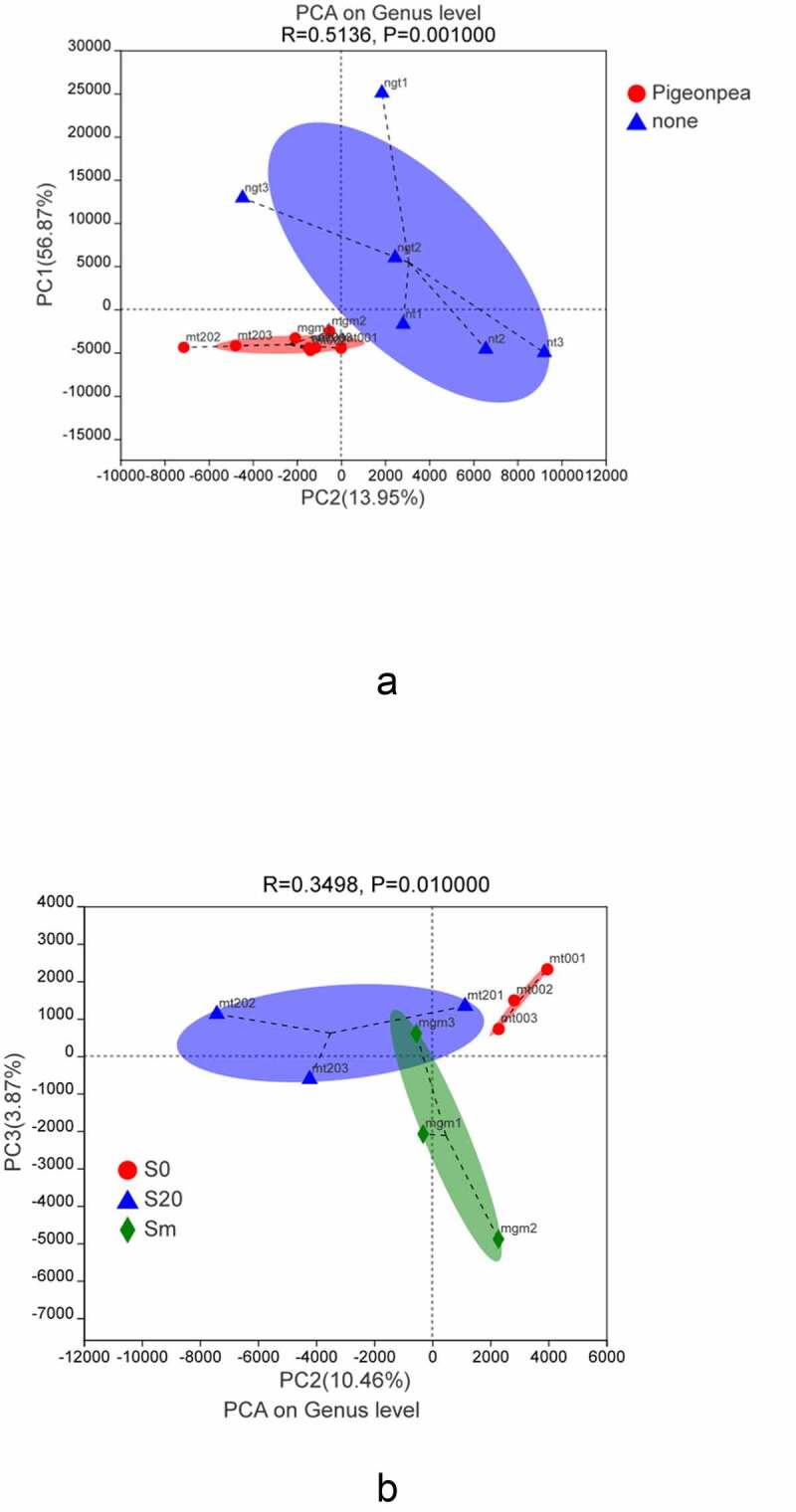
A) Principal component analysis of bacteria in cultivated and unplanted soil. B) Principal component analysis of soil bacteria at different depths of soil planted with *C. cajan.*

To determine the elements and structure, the main data were processed to remove noise and redundancies; the original complex data dimension was reduced to reveal the simple structure hidden behind the complex data obtained through the PCA of the soil samples. Figure 6a shows that planting *C. cajan* had a significant effect on the bacterial community and the interpretation degrees of the PC1 and PC2 axes were 56.87% and 13.95%, respectively. Figure 6b represents the different depths of the soil samples in different layers. As can be seen from the figure, there were significant differences in the bacterial compositions of the soil samples obtained from different depths. The explanatory degrees of PC2 and PC3 axes were 10.46% and 3.87%, respectively.

### 3.6 Species association analysis

**Figure 7.**
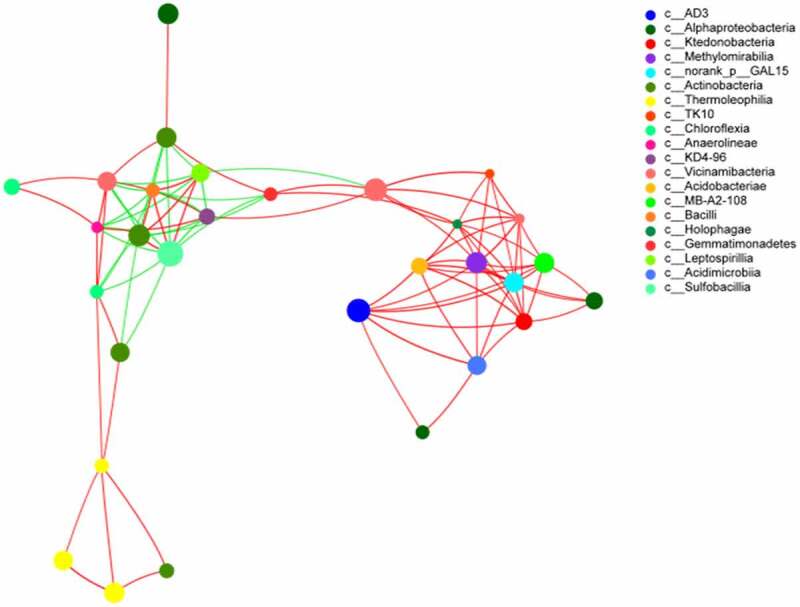
Network correlation network analysis of soil at different depths

To determine the correlation between the soils at different depths for species analysis, 20 species at the class level were selected. The total abundance, species, and Spearman’s correlation coefficient between the calculated results are shown in Figure 7. The size of the nodes in the graph reflects the species abundance, different colors represent different species, and the color of the attachment indicates a positive (red) or negative (green) correlation. The thickness of the line indicates the correlation coefficient: the thicker the line, the higher is the correlation between species. In addition, more the number of lines, the more closely related the species is to other species. The clustering coefficients of *Xanthobacteraceae, G_methylocystis, G_Streptomyces, G_Norank_f_Roseiflexaceae*, and *G_Gaiella* were the largest; the clustering coefficient value was 1, which indicated that these bacteria are the most important among network nodes. The clustering coefficient of *G_Sphingomonas* was 0, which indicated that this node has almost no relationship with its neighboring nodes.

To evaluate the obtained community abundance data, the Kruskal–Wallis *H* test was performed on species present among the microbial colonies at the different depths of soil planted with *C. cajan*. Using strict statistical methods, differences in the significance level of species abundance at the genus level were evaluated and a list of significantly different species among different groups was obtained. The *p* < 0.01 of *Sulfobacillus* and *Acidimicrobium* showed significant difference at different soil depths, and the abundance in the mixed-layer soil was significantly higher than that of the other two groups [25]. The *p* < 0.05 of *norank_f_norank_o_Vicinamibacterales, Nocardioides, Arthrobacter, norank_f_Vicinamibacteraceae* also differed with soil depth [26], whereas *Acidothermus* and *Gaiella* showed no significant difference (p > 0.05). *Sulfobacillus* can reduce Fe^3+^ under anaerobic conditions and oxidize Fe^2+^ and sulfide ore under aerobic conditions [27]. The mixed-layer soil is a mixture of coal gangue and covering soil. Coal gangue contains pyrite, accounting for approximately 5% [28], and long-term stacking causes great harm to the environment. However, sulfobacilli in soil oxidizes pyrite and can produce sulfate minerals. *Acidimicrobium* can catalyze the oxidation of other ions (such as fluorine, silver, and iron) and plays an important role in the biological restoration of the environment [29]. Vicinamibacteraceae are aerobic, neutrophilic, and psychrotolerant to mesophilic chemoheterotrophs. *Nocardioides* can degrade riboflavin and lumichrome as carbon sources [30]. *Arthrobacter* is another bacterium that is beneficial in environmental pollution control. 31,reported that *Arthrobacter* can degrade polycyclic aromatic hydrocarbons in petroleum pollutants. The abundance of these bacteria in group S0 was significantly higher than that in the other two groups, which was attributed to their aerobic nature; furthermore, their content decreased with increasing soil depth.

### 3.7 Functional analysis of microflora

**Figure 8.**
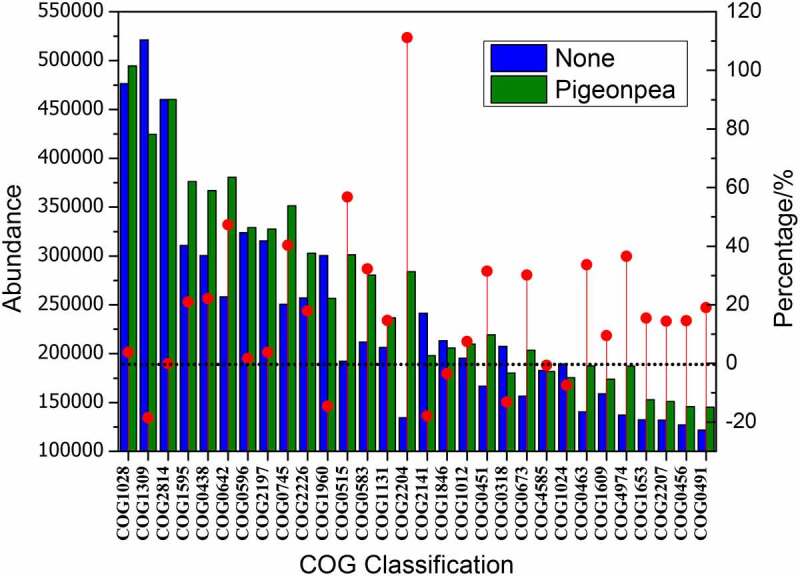
Functional analysis of microflora

The abundance table of OTUs was standardized by PICRUSt; the influence of the copy number of 16S marker genes in species genomes was removed. The COG function annotation of OTUs performed by greengene ID corresponded to each OTU, and the annotation information of OTUs at COG function level and abundance information of each function in different samples were obtained (Figure 8, only COG with the top 30 abundance is displayed). As can be seen in Figure 8, planting *C. cajan* had a significant influence on the function of microflora. In the soil planted with *C. cajan*, the abundance of 22 enzymes corresponding to COG in the top 30 COG increased. Among them, COG2204 represents the OTU abundance of sigma 54-specific transcriptional regulator, which increased by 111.2%. In addition, COG0642 (histidine kinase), COG0745 (histidine kinase), and COG0515 (serine/threonine protein kinase) increased by 47.4%, 40.3%, and 47.4%, respectively. COG1028, COG1012, COG0673, COG0491, and COG0456, which represent dehydrogenase reductase, also increased by 10%–30%. COG1309, COG2141, and COG1024, which represent transcriptional regulators, monooxygenase, and acyl-CoA dehydrogenase, decreased by 18.6%, 17.9%, and 7.4%, respectively.

There were obvious differences in enzyme abundance at different soil depths. Sulfite oxide plays a vital role in the process of sulfur metabolism and sulfur detoxification in plants [32]. The abundance of sulfite oxide in surface S0, deep S20, and the gangue mixed layer (Sm) was 299, 93, and 312, respectively, and was the highest in the mixed layer, which may be related to sulfide minerals in gangue.

Catalase peroxidase is related to respiration, photosynthesis, and auxin oxidation and widely exists in plants [33]. The abundance of catalase peroxidase was the highest in the surface soil (S0). Nitrogenase can reduce nitrogen molecules to ammonia and is therefore an important enzyme for nitrogen fixation in plants. In the deep soil (S20), the highest abundance of nitrogenase was 5865, which is 3.3 times that of the surface abundance.

D-cysteine desulfurases utilize a PLP-dependent mechanism to catalyze the conversion of D-cysteine to D-alanine and sulfane sulfur through the formation of a protein-bound cysteine persulfide intermediate on a conserved cysteine residue [34]. Cysteine desulfurases participate in the biosynthesis of thio-cofactors, including Fe–S clusters, thionucleosides, DNA sulfur modifiers, thiamin, and molybdenum cofactor [35]. D-cysteine desulfurases in the soil planted with *C. cajan* were decreased (4657 in the surface soil and 2675 in the mixed soil of coal and gangue). Nitrogen monoxide is an inevitable intermediate product in the denitrification process and is also a cytotoxin [36,37]. If a large amount of accumulation threatens the life of cells, nitric-oxide reductase can reduce nitric oxide to nontoxic nitrous oxide, which plays an essential role in biological nitrogen removal [43]. Nitric-oxide reductase abundance was highest in the mixed layer. The analysis of enzyme abundance in soil samples is beneficial to explore the interaction between soil and *C. cajan*. Therefore, it is a crucial guiding tool for ecosystem restoration via soil microorganisms in coal gangue.

This study showed that planting *C. cajan* is a highly effective method for restoring the waste coal gangue dump; it can effectively prevent soil erosion and inhibit the diffusion of pollutants (such as heavy metals). Moreover, planting *C. cajan* affected the composition of soil microorganisms, which was also influenced by soil depth. Future research should aim to isolate differential microorganisms from different sources of soil so that their metabolic pathways can be studied in detail. Of note, soil microorganisms can promote plant growth in waste gangue dump reclamation.

## 4. Conclusion

In the samples of soil that were planted with *C. cajan*, the relatively abundant microflora included *Actinobacteria*, α-*Proteobacteria, Acidimicrobiia, Thermoleophilia*, and others, whereas in unplanted soil samples, the abundant microflora mostly comprised *Actinobacteria*. The *p* value of *Sulfobacillus* and *Acidimicrobium* significantly differed at different soil depths, and the abundance in mixed-layer soil was significantly higher than that in the other two groups. The abundance of sulfite oxide in surface S0, deep S20, and mixed-layer Sm was 299, 93, and 312, respectively, and thus was the highest in the mixed layer, which may be related to sulfide minerals in gangue.

## Article highlights

- Microbial species at different soil depths were identified.
- Effects of planting *C. cajan* in soil microbial succession were presented.
- Enzyme abundance was analyzed to explore the interaction between soil and *C. cajan*.
